# Editorial: Consciousness, cognition, and compassion

**DOI:** 10.3389/fpsyg.2022.998315

**Published:** 2022-09-07

**Authors:** Balachundhar Subramaniam, Tracy F. H. Chang, Senthilkumar Sadhasivam

**Affiliations:** ^1^Harvard University, Cambridge, MA, United States; ^2^Department of Anesthesia, Beth Israel Deaconess Medical Center, Harvard Medical School, Boston, MA, United States; ^3^Department of Labor Studies and Employment Relations, School of Management and Labor Relations, Rutgers, The State University of New Jersey, Piscataway, NJ, United States; ^4^Department of Anesthesiology and Perioperative Medicine, University of Pittsburgh Medical Center, Pittsburgh, PA, United States

**Keywords:** Inner Engineering, PPE, agency, EEG, consciousness, cognition, compassion

COVID-19 has rapidly disrupted how we live on multiple levels in an unprecedented scale globally. As of May 15, 2022, 525 million people worldwide have contracted the coronavirus, and approximately 6.25 million people have succumbed to the disease. In addition, the massive disruptions of life—loss of loved ones and employment, living with long-term debilitating conditions, shortage of personal protective equipment (PPE), closures of businesses and schools, exposure to trauma, and social isolation, have triggered severe symptoms of mental distress and illness. As of June 2020, 31% of surveyed respondents self-reported anxiety or depression, 13% reported increased substance use, 26% reported stress-related symptoms, and 11% reported having serious thoughts of suicide in the past 30 days (https://www.nimh.nih.gov/about/director/messages/2021/one-year-in-covid-19-and-mental-health, accessed October 20, 2021). These issues have risen so much that an editorial in the New England Journal of Medicine cautioned about a parallel mental health pandemic (Pfefferbaum and North, 2020).

Although susceptible to environmental influences, humans have the capacity to exercise agency—people are agents of influencing themselves as well as influencing their environment to produce self-generated influences (Bandura, 1989). People can act as agents over their environment by drawing on their knowledge, cognitive, and behavioral skills to obtain desired results; moreover, people can act as agents over themselves by monitoring their actions, mobilize cognitive resources, and motivating themselves to bring about desirable personal change (Bandura, 1989). This exercise of human agency is evident during the COVID-19 pandemic. Scientists have raced to develop vaccines against COVID-19, and government agencies have implemented vaccination at a historic speed – 4.85 billion people have chosen to be fully vaccinated (62% of the world population) as of July 16, 2022. Declining confirmed cases, vaccination, availability, use of personal protective equipment (PPE), and a weaker COVID-19 variant have eased travel restrictions and social distancing and enabled us to return to our “normal” life. Physical PPEs and vaccination may protect us from physical harm caused by the virus, but they may not be sufficient to protect us from psychological distress. We need a different kind of PPEs – Inner PPEs. The ancient science of yoga prescribes these inner tools and methods by mobilizing our physiological, cognitive, and emotional capacities that could buffer the psychological impact of COVID-19.

According to Lazarus and Folkman (1984) and Kabat-Zinn (2013) the meaning that we give to an event and our capability to mobilize inner resources determine whether a situation is stressful or not. In recent years, an exponential amount of research has shown that yoga and meditation are effective methods for reducing mental stress and physical ailments and experiencing greater physical and psychological wellbeing (Hendriks et al., 2017; Domingues, 2018; Goldberg et al., 2022). However, yoga and meditation interventions have been heterogeneous, varying in types of yoga (e.g., physical postures or *asanas*, breath work or *pranayama*, and meditation), duration, and format (in-person vs. online) (Hendriks et al., 2017; Domingues, 2018; Matko and Sedlmeier, 2019).

The Research Topics collection introduces a yogic methodology that is less known to western researchers but has begun to attract scientific evaluation on its effect on physical and mental wellbeing before the COVID-10 pandemic. This methodology is called “Inner Engineering” (Sadhguru, 2016), which consists of a system of “technologies for wellbeing” derived from the science of yoga (https://www.innerengineering.com, accessed July 16, 2022). This system of technologies integrates four branches of yoga – knowledge (*Jñāna Yoga)*, emotion (*Bhakti Yoga*), physical action or body *(Karma Yoga)*, and energy *(Kriya Yoga)*. These “technologies” engineer one's thoughts, emotions, postures, and energy so that wellbeing can be maintained and psychological distress mitigated, especially during disruptions and difficulties. The system consists of multiple components and different levels of practices and programs designed for absolute beginners to advanced practitioners. The beginner practices include Upa Yoga, Isha Kriya, and Simha Kriya, which can be taught and learned online. Chang et al. (2022a) found that Upa Yoga reduced stress and increased wellbeing among college students during the COVID-19 pandemic.

Breathing is consistently affected from the beginning to the late stages of contracting the coronavirus. A significant number of patients following COVID-19 had lasting breathlessness, brain fog, and fatigue, collectively called Long-COVID. In their conceptual paper titled “Can Yogic Breathing Techniques Like *Simha Kriya* and *Isha Kriya* Regulate COVID-19-Related Stress,” Rain et al. proposed that certain yogic breathing techniques, such as Simha Kriya, and meditative practices, such as Isha Kriya, have a basis to help these patients consistently. They explained that these breathing techniques give a better lung condition and can protect from inflammation. This hypothesis remains to be tested for COVID-19 patients. Narayanan et al. (2021) showed that Simha Kriya was feasible and acceptable among healthcare workers during the COVID-19 pandemic.

The core program of the “Inner Engineering” methodology is Inner Engineering Online (IEO), which consists of 7 online lessons and a system of Upa Yoga. Studies showed that IEO has a positive effect on employee wellbeing (mindfulness, joy, vitality, improved sleep quality), positive work experiences (a higher level of psychological capital, meaningful work, and work engagement) (Chang, 2020, 2021; Chang et al., 2022b), and self-leadership (Chang et al., forthcoming). Upadhyay et al. (2022) found that IEO reduced stress among technology professionals.

After completing the IEO, one can participate in Inner Engineering Completion (IEC), a 2-day in-person program before the COVID pandemic. During COVID, the IEC program has been converted to an online format. The Inner Engineering Completion online Program reduced stress and enhanced wellbeing, thus providing similar effects to that of a face-to-face IEC (Upadhyay et al.). The IEC is one of the prerequisites for more advanced programs, such as Samyama. In the paper titled “Advanced Meditation Alters Resting – State Brain Network Connectivity Correlating with Improved Mindfulness,” Vishnubhotla et al. investigated the effect of an intensive 8-day Samyama meditation program (an advanced “Inner Engineering” program) on the brain functional connectivity using resting-state functional MRI and self-reported mindfulness scores. Their proof-of-concept work showed that after the intensive 8-day Samyama meditation retreat, participants had an increased resting-state functional connectivity between the salience and default mode networks. During focused breath watching, participants had lower intra-network connectivity in specific networks. Increased connectivity correlated with an improved self-reported mindfulness score. Their work showing the relationship between network connectivity and executive function is important and needs to be replicated in a larger sample size.

In another paper titled “Isha Yoga Practices and Participation in Samyama Program are Associated with Reduced HbA1C and Systemic Inflammation, Improved Lipid Profile, and Short-Term and Sustained Improvement in Mental Health: A Prospective Observational Study of Meditators,” Sadhasivam et al. studied the same 8-day retreat with a sample of 632 adults. The most impressive result was a sustained reduction in depression or anxiety after 3 months of this retreat. Positive psychological measures such as vitality, resilience, and joy were increased post-retreat and sustained at 3 months. There was a reduction in their HbA1C with dietary restriction in their preparatory phase. Most importantly, they had lower CRP levels at all-time points than the controls, indicating a sustained benefit from the preparatory Inner Engineering practices they were doing before the retreat. These conditions would give the body a better state if COVID-19 infection happened in these participants.

In addition to featuring studies on Inner Engineering practices, this issue highlights one Perspective article titled “Etiology of Burst Suppression EEG Patterns” and one Review article titled “Cognition and Pain: A Review.” EEG descriptions of meditative states exist for different techniques. Meditative states may have the ability to take someone to burst suppression or, at the very least, low-frequency states. Creating burst suppression medically for an injured brain is well described. However, burst suppression in a non-injured brain is questionable and not recommended in the perioperative period. The various etiology for burst suppression states are explored by Shanker et al. Meditation is known to increase pain tolerance and enhance cognition. In the perioperative setting, enhanced pain can lead to altered cognitive states such as delirium. Thus, meditation can be used as an adjunct in the perioperative period to reduce the pain and enhance cognition. In their article “Cognition and Pain: A Review,” Khera and Rangasamy expand on what we know about cognition and pain.

The research on the “Inner Engineering” methodology derived from the science of yoga is in its infancy and faces certain limitations and challenges. One challenge is the adherence rate – how to define adherence, level of adherence, and predictors of adherence (Beatty and Binnion, 2016; Musiat et al., 2022). The adherence rates to behaviorally based interventions range from 20 to 80%, depending on the definitions and pre-screening. The adherence rate particularly low for online intervention – about 50% for online mindfulness programs (Winter et al., 2022). Another challenge is research methodology. The current body of work published in this collection has addressed some of these issues.

Consciousness is a subjective experience. Many yogis describe the loss of time, space, and bodily sensations. How do we capture these subjective experiences and study them? Neuroscience focuses on the manifestations of consciousness (e.g., cognitive and brain activities) (Melloni et al., 2021). Chalmers (2007) classified these “objective functions” as the “easy problem” of consciousness. The “hard problem” of consciousness is the *subject's conscious experience* that cannot be explained by physical account (Chalmers, 2007; Shanta, 2015).

In conclusion, this issue summarized the importance of understanding the science of yoga in the form of the less known Inner Engineering methodology and its ability to support our physical and mental wellbeing while we face unprecedented challenges posed by the COVID-10 pandemic. The issue marks the beginning of growing research programs on this methodology and extends the frontier of research on the science of yoga and meditation and its impact on consciousness, cognition, and compassion.

## Author contributions

All authors listed have made a substantial, direct, and intellectual contribution to the work and approved it for publication.

## Conflict of interest

The authors declare that the research was conducted in the absence of any commercial or financial relationships that could be construed as a potential conflict of interest.

## Publisher's note

All claims expressed in this article are solely those of the authors and do not necessarily represent those of their affiliated organizations, or those of the publisher, the editors and the reviewers. Any product that may be evaluated in this article, or claim that may be made by its manufacturer, is not guaranteed or endorsed by the publisher.
